# Detection of non-travel-associated, ceftriaxone non-susceptible *Neisseria gonorrhoeae* FC428-like harbouring the mosaic *penA60* allele in Ontario, Canada

**DOI:** 10.14745/ccdr.v51i101112a06

**Published:** 2025-12-12

**Authors:** Adam S Komorowski, Alireza Eshaghi, Jennifer Burbidge, Karen Johnson, Andrea Saunders, Austin Zygmunt, Maan Hasso, Huda Almohri, Irene Martin, Samir N Patel, Vanessa Tran

**Affiliations:** 1 Department of Pathology and Molecular Medicine, McMaster University, Hamilton, ON; 2 Division of Infectious Diseases, Department of Medicine, McMaster University, Hamilton, ON; 3 Michael G. DeGroote Institute for Infectious Disease Research, McMaster University, Hamilton, ON; 4 Research Institute of St. Joseph’s Healthcare Hamilton, Hamilton, ON; 5 Department of Health Research Methods, Evidence, and Impact, Faculty of Health Sciences, McMaster University, Hamilton, ON; 6 Public Health Ontario, Toronto, ON; 7 Department of Family Medicine, Faculty of Medicine, University of Ottawa, Ottawa, ON; 8 Department of Laboratory Medicine and Pathobiology, Faculty of Medicine, University of Toronto, Toronto, ON; 9 LifeLabs, Etobicoke, ON; 10 Infectious Diseases and Medical Microbiology, William Osler Health System, ON; 11 National Microbiology Laboratory, Public Health Agency of Canada, Winnipeg, MB

**Keywords:** gonorrhea, *Neisseria gonorrhoeae*, sexually transmitted diseases, drug resistance, public health surveillance

## Abstract

**Background:**

This case report describes a young male with multidrug-resistant *Neisseria gonorrhoeae* infection acquired in Ontario, Canada with no travel history.

**Methods:**

Case follow-up was conducted following routine public health practice in Ontario. Antimicrobial susceptibility testing of the isolate was done by agar dilution. Strain typing and other molecular characterization was done by whole genome sequencing.

**Results:**

The patient was treated successfully with intramuscular ceftriaxone and oral azithromycin. Agar dilution testing demonstrated reduced susceptibility to all tested agents, except for azithromycin and spectinomycin, including non-susceptibility to ceftriaxone (minimum inhibitory concentration [MIC]=0.5 mg/L) and cefixime (MIC=2 mg/L), resistance to tetracycline (MIC=2 mg/mL) and ciprofloxacin (MIC=32 mg/L), and testing intermediate to penicillin (MIC=1 mg/L). Whole-genome sequencing revealed the isolate was closely related to the FC428 clone, which harbours the mosaic *penA60* allele responsible for elevated MICs to extended-spectrum cephalosporins, such as ceftriaxone or cefixime, both currently recommended as first-line or alternative treatment options for uncomplicated anogenital gonorrhea infections in Ontario.

**Conclusion:**

Identification of this case suggests previously unrecognized local transmission of this multidrug-resistant *N. gonorrhoeae* strain is occurring in Ontario and highlights the need for ongoing surveillance to monitor trends and inform treatment recommendations.

## Introduction

*Neisseria gonorrhoeae* (*N. gonorrhoeae*) is the second most commonly reported sexually transmitted infection in Canada ((1)), with 92.34 cases per 100,000 population in 2022, representing an increase of 175.9% since 2010 ((2,3)). Infection rates among both males and females have increased over time, though more rapidly in males ((2)). While the highest rate of gonorrhea infections remains in the 20–29 age group in Canada, the greatest relative increases between 2010–2019 were identified in those 30–39 years old and 40–59 years old ((2)).

*Neisseria gonorrhoeae* antimicrobial resistance has increased over the past 20 years and is a public health concern, as untreated, and untreatable, gonorrhea poses a significant risk of reproductive morbidity and can increase susceptibility to HIV transmission and acquisition ((4,5)). The ability of *N. gonorrhoeae* to develop resistance to antimicrobials is due to a combination of transferrable plasmid-borne resistance determinants, as well as chromosomal genes that result in antimicrobial destruction, target modification, decreased membrane permeability to antimicrobials, or drug efflux ((6)).

*Neisseria gonorrhoeae* FC428 has been implicated in multiple clonal outbreaks of gonorrhea infection in Asia, Europe, and the United Kingdom ((7–12)). This article reports the first known case of ceftriaxone non-susceptible *N. gonorrhoeae* FC428 infection with the mosaic *penA60* allele identified in an Ontario patient. Notably, the patient lacked a compatible travel history typically associated with FC428 infection, suggesting under-recognized local transmission.

## Methods

### Case presentation

A young adult male who reported a risk factor of having condomless sex with the opposite sex, presented to a walk-in clinic in Ontario with a one-week history of dysuria and urethral discharge. The patient denied any urinary urgency, persistent sore throat, or neck swelling. He had a history of one episode of unprotected insertive vaginal intercourse with a female partner (not disclosed to be a sex professional) two weeks prior to symptom onset. He was unable to recall whether insertive oral intercourse also occurred. The patient denied any recent travel outside of Ontario.

Following counselling, a first-void urine specimen and a urethral swab were aseptically obtained, the latter being placed into Amies with charcoal transport media. The patient was treated empirically with ceftriaxone 250 mg given as a single intramuscular dose in the gluteal muscle, as well as azithromycin 1g given as a single oral dose. Nucleic Acid Amplification Test (NAAT) by strand displacement amplification on the urine specimen was performed using the BD Viper^TM^ (Becton, Dickinson and Company), which was positive for *N. gonorrhoeae* and negative for *Chlamydia trachomatis*. The positive *N. gonorrhoeae* was confirmed by polymerase chain reaction (PCR) using the BD MAX™ platform.

A presumptive isolate of *N. gonorrhoeae* from the urethral swab was submitted to Public Health Ontario (PHO), which is a provincial public health reference laboratory, for confirmatory and antimicrobial susceptibility testing. The laboratory performed *N. gonorrhoeae* culture by plating specimens on New York City agar and incubating at 35–37°C in 5% carbon dioxide for 48 hours. Matrix-Assisted Laser Desorption/Ionization Time of Flight Mass Spectrometry (MALDI-TOF MS) was used to confirm the identity of any oxidase-positive colonies, alongside cysteine trypticase agar carbohydrate utilization and O-Nitrophenyl-β-D-galactopyranoside (ONPG) testing. The isolate was oxidase positive, ONPG negative, and utilized dextrose but not maltose or sucrose, consistent with *N. gonorrhoeae*. Susceptibility testing was performed on the *N. gonorrhoeae* isolate using the agar dilution method recommended by the Clinical and Laboratory Standards Institute (CLSI) and interpreted using the CLSI breakpoints ((13,14)). The isolate was sent to Canada’s National Microbiology Laboratory (NML) for confirmatory agar dilution testing. Minimum inhibitory concentrations (MICs) of the clinical isolate are shown in **Table 1****.**

**Table 1:** Antimicrobial susceptibility testing results for *Neisseria gonorrhoeae* patient isolate

Antimicrobial tested	Minimum inhibitory concentration (MIC), mg/L	Interpretation^a^
Penicillin	1	Intermediate
Ceftriaxone	0.5	Non-susceptible
Cefixime	2	Non-susceptible
Ertapenem	0.06	N/A
Azithromycin	0.25	Susceptible
Gentamicin	8	N/A
Tetracycline	2	Resistant
Ciprofloxacin	32	Resistant
Spectinomycin	16	Susceptible

Abbreviation: N/A, no breakpoint available

^a^ Clinical Laboratory Standards Institute, MIC breakpoint interpretations as per Clinical and Laboratory Standards Institute (CLSI) M100, 33^rd^ edition. Note that the MICs for ceftriaxone and cefixime are considered resistant using the European Committee on Antimicrobial Susceptibility Testing (EUCAST) breakpoints (Version 14.0)

The patient returned to the walk-in clinic 18 days after completion of treatment for follow-up and reported no symptoms indicative of treatment failure. A urethral swab was obtained for test-of-cure by culture and tested negative. The patient was discharged from follow-up. The patient did not disclose his sexual contact for public health follow-up; he was advised to let the contact know to seek testing and treatment for suspected gonorrhea.

## Results

### Whole-genome sequencing and molecular typing

**Figure 1:**
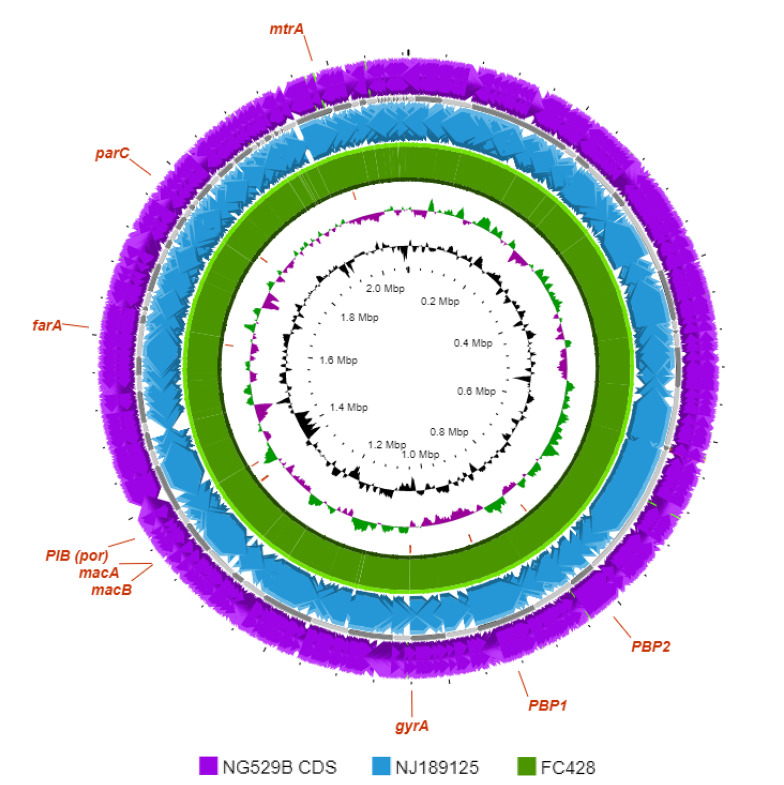
Graphical display of distribution of genome annotations Legend: Circular map showing a comparison of the genome of *Neisseria gonorrhoeae* strain NG 529B together with closely related genomes using Proksee. The outermost ring represents the NG 529B chromosome position, the grey-coloured ring represents the genome backbone (in contigs), the blue ring represents strain NJ189125, and the green ring represents strain FC428 (NZ_AP018377.1). The GC Skew (purple/green) is represented as the second innermost ring. The GC content is represented as the innermost ring (black). This whole genome shotgun project has been deposited at DDBJ/ENA/GenBank under the accession JBHFAD000000000. The version described in this paper is version JBHFAD010000000

Whole-genome sequencing was used to elucidate the genetic markers responsible for the resistance profile of the patient’s isolate. Genomic DNA was extracted using the EMAG® system (bioMérieux SA, Marcy-l’Étoile, France) and performed sequencing on the MiSeq instrument (Illumina Inc., San Diego, United States). Raw FASTQ files were assembled using CLCGenomics Workbench version 8.5.3 (CLC bio, Germantown, Maryland, United States), and the assembled genome was submitted to the Bacterial and Viral Bioinformatics Resource Centre (BV-BRC) ((15)) for genome annotation and comparison. The assembled genome was of good quality overall, with 88 contigs, a total length of 2.129 Mb, an average guanine-cytosine content of 52.37%, and an average coverage of 641x. **Figure 1** shows a graphical display of the genome annotation.

Antimicrobial resistance determinants were identified in the genome using the Comprehensive Antibiotic Resistance Database (CARD) ((16)) to assign a functional annotation and broad mechanism of resistance, where possible. The resistance determinants identified are listed in **Table 2**, and include genes responsible for target alteration, target protection, reduction of cell wall permeability, and the production of efflux pumps. Of note, the patient’s isolate harbours the mosaic *penA60* allele, which has the *penA* A311V mutation and is associated with increased MICs to cephalosporins ((17)). In silico analysis was largely concordant with antimicrobial susceptibility testing.

**Table 2:** Antimicrobial resistance genes identified from sequenced *Neisseria gonorrhoeae* patient isolate according to the Comprehensive Antibiotic Resistance Database

RGI criteria	Antimicrobial resistance gene	Single nucleotide polymorphism(s)	Antimicrobial classes affected	Resistance mechanism	% Identity of matching region	% Length of reference sequence
Perfect	mtrA	mtrR-promoter:g.-57_-57del, and mtrR-promoter:p.H105Y	Macrolide antibiotic, penam	Efflux	100	100
Strict	PBP1 (ponA)	L421P	Cephalosporin, cephamycin, penam	Target alteration	97.24	100
Strict	PBP2 (penA) mosaic	A311V, V316T, I312M, T483S, F504L, A510V, N512Y, H541N, I515V, G545S, I566V	Cephalosporin, cephamycin, penam	Target alteration	91.58	100.17
Strict	rpsJ	V57M	Tetracyclines	Target protection	99.03	100
Strict	porin PIB (porB)	G120K, A121D, I218M, A323V, M18T, Q143K, M257T, G259V, S258R, N297D	Monobactam, carbapenem, cephalosporin, cephamycin, penam, tetracycline antibiotic, penem	Reduced permeability	96.26	100
Strict	parC	S87R, V596I	Fluoroquinolone antibiotic	Target alteration	99.74	100
Strict	gyrA	S91F/D95A	Fluoroquinolone antibiotic	Target alteration	99.67	100
Strict	mtrC	mtrC-promoter:p.G29R and S163G	Macrolide antibiotic, penam	Efflux	95.63	100
N/A	folP^a^	P68S, R228S	N/A	N/A	N/A	N/A
N/A	rpoB^a^	H552N	N/A	N/A	N/A	N/A

Abbreviations: N/A, not applicable; RGI, resistance gene identifier

^a^ Insufficient data were present in the Comprehensive Antibiotic Resistance Database (CARD) database to assign interpretations to the single nucleotide polymorphisms identified for this antimicrobial gene

Note: The CARD database was accessed March 2023

*Neisseria gonorrhoeae* multilocus sequence typing (MLST), multi-antigen sequence typing (NG-MAST), and *N. gonorrhoeae* Sequence Typing for Antimicrobial Resistance (NG-STAR) were confirmed using contigs obtained by *de novo* assembly via pubMLST. The isolate was assigned a sequence type of ST13943 using MLST, and clonal complex 233 in NG-STAR. This patient’s isolate contained a novel porB-2035 and *tbpB*-21 allele combination, which was assigned as ST-21711 by NG-MAST v.2.0.

**Figure 2:**
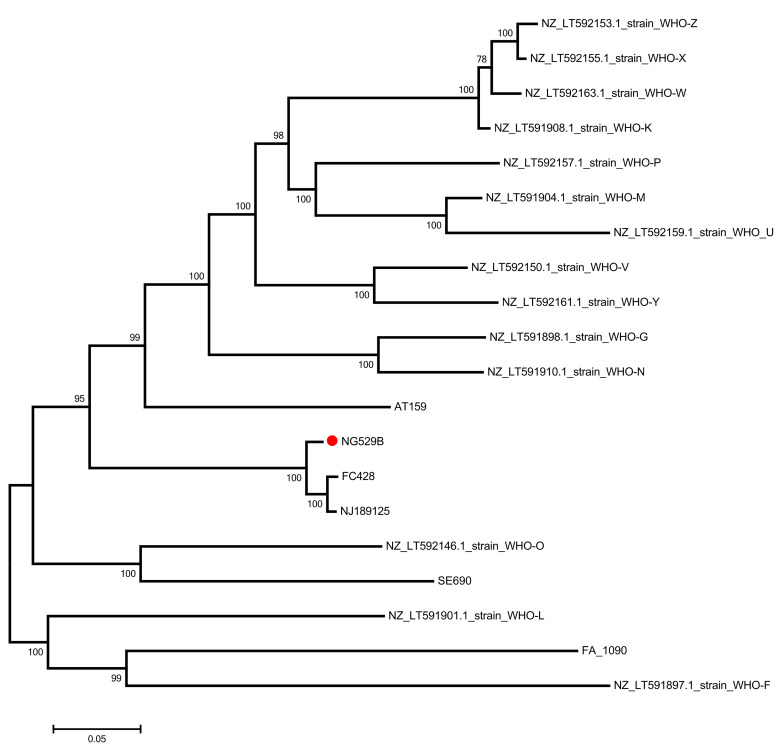
Phylogenetic tree of *Neisseria gonorrhoeae* patient isolate Legend: Maximum likelihood phylogenetic tree based on core-genome single nucleotide polymorphisms (SNPs) across World Health Organization strains and recently reported strains harbouring the mosaic *penA60.001* gene and the strain from this study (red dot, ‘NG529B’). The tree was constructed using 9116 SNPs and drawn to scale, with branch lengths measured in the number of substitutions per site

Finally, to examine the relatedness of the Ontario isolate to other resistant strains, phylogenetic analysis was performed and a maximum-likelihood phylogenetic tree was created using 9,116 core-genome single nucleotide polymorphisms (SNPs) across *N. gonorrhoeae* World Health Organization (WHO) strains and strains reported to harbour the mosaic *penA60* allele (**Figure 2**). The phylogenetic tree illustrates the similarity of the patient isolate’s genome to that of clone FC428.

## Discussion

*Neisseria gonorrhoeae* infection is an important cause of morbidity and is transmitted via mucosal contact during oral, vaginal, or anal intercourse, as well as vertically during childbirth ((6)). It is an important cause of cervicitis in females and urethritis in males, though upwards of 50% of females remain asymptomatic ((6)). In Canada, gay, bisexual, and other men who have sex with men (GBMSM) are a key population at risk of infection ((2)). Ascending, untreated infection can be a cause of infertility and other urogenital complications. Over the past decade, gonorrhea infection and resistance rates have increased at an alarming pace ((2)). However, because only a small percentage of gonorrhea cases are identified with culture, prevalence estimates of multidrug resistance may be underreported. Combating the spread of *N. gonorrhoeae*, in the absence of effective vaccination and poor uptake of chemoprophylaxis, requires a multifaceted approach consisting of sexual health education, use of barrier protection, adequate surveillance infrastructure, timely access to testing and treatment, and an effective contact notification strategy ((6)).

This case report describes the first known ceftriaxone non-susceptible *N. gonorrhoeae* FC428-like isolate harbouring the mosaic *penA60* allele in Ontario, Canada, that was not linked to travel. This was the third ceftriaxone-non-susceptible strain of *N. gonorrhoeae* identified in Ontario and the first detected since 2018. Single nucleotide polymorphism (SNP) analysis demonstrated that the patient’s isolate was most closely related to *N. gonorrhoeae* clone FC428, a strain first recognized in Nanjing, China in 2018. Similar to our patient’s isolate, clone FC428 harbours the mosaic *penA60* gene and has elevated MICs to ceftriaxone and cefixime. While the mosaic *penA60* allele has been previously identified in patients from the Canadian provinces of Québec and Alberta and may be associated with an increased risk of treatment failure ((18,19)), this is the first instance of its identification in Ontario—this allele is a growing concern worldwide, having been isolated in Asia, Australia, Europe, and North America ((7,10,18–23)). Of note, although the first two cases in Ontario did not have the *penA60* allele, they did have the *penA* A311V mutation of interest. Interestingly, this case had clinical resolution despite phenotypic non-susceptibility to cephalosporins; however, as the proportion of resistant isolates increases over time, it may be worthwhile to consider use of a higher dose of ceftriaxone as standard of care for uncomplicated gonorrhea infections. Of note, in December 2024, the Public Health Agency of Canada updated its treatment guideline to recommend 500 mg ceftriaxone monotherapy as the preferred treatment of uncomplicated gonorrhea infections for adults ((24)). Although some mutations associated with elevated macrolide MICs were detected, it was not enough to confer azithromycin resistance (the isolate was phenotypically susceptible), since resistance is mediated through additive effects of multiple mutations within the multidrug efflux pump system (mtrCDE) operon. Clinical cure in this patient’s case may have been related to azithromycin administration.

### Limitations

Identification of this isolate suggests that transmission of ceftriaxone non-susceptible *N. gonorrhoeae* is occurring in Ontario. As of the time of publication, four additional ceftriaxone non-susceptible *N. gonorrhoeae* isolates have been identified in the province that bore different MLST and NG-MAST types than the case described. As *N. gonorrhoeae* case numbers and antimicrobial resistance rapidly increase, there is an urgent need for renewed public health messaging and guidance to help reduce the transmission rate in Canada.

An increasing incidence of multidrug-resistant gonorrhea infection in Canada should incentivize public health laboratories to create new strategies to rapidly identify whether treatment failures or suspected outbreaks may be clonal in nature. This approach has been adopted by the NML ((25,26)) and the United Kingdom’s Health Security Agency, with the implementation of a mosaic *penA60* allele PCR test, specifically designed to detect the *penA* A311V mutation ((19,20)). Another recent protocol developed in China uses a multiplex high-resolution melting assay to genetic markers of resistance to cephalosporins and azithromycin ((27)). In a cross-sectional study, when compared to phenotypic testing, this assay was shown to have a specificity of 96.29% (95% CI: 94.57–97.50) for cefixime and 99.52% (95% CI: 98.68–99.85) for azithromycin ((28)). Of note, the assay’s sensitivity was significantly lower for both ceftriaxone (79.10%, 95% CI: 63.52–89.42) and azithromycin (31.34%, 95% CI: 20.87–43.97) ((28)). The identification of this case also highlights the continued relevance of culture-based diagnostics at local laboratories in Canada, in the absence of widespread NAAT that includes predominant genetic markers of resistance. As treatment becomes more challenging for gonorrhea, enhanced uptake of emerging preventive interventions, such as doxycycline post-exposure prophylaxis, may need to be considered.

## Conclusion

This case highlights the growing threat of multidrug-resistant *N. gonorrhoeae* infections, which are being increasingly identified in Canada ((18,19)). It underscores that laboratory surveillance programs should include a combination of genotypic and phenotypic testing methods to help investigate isolates with multidrug resistance and treatment failures and highlights the continued relevance of culture-based diagnostics. However, as diagnostics continue to shift from culture-based to molecular-based methods, it is important to continue exploring direct testing of NAAT specimens for antimicrobial resistance prediction. Healthcare professionals should screen for gonorrhea as per national guidelines ((29)) and test individuals with compatible signs and symptoms in order to identify and treat those with gonorrhea and reduce *N. gonorrhoeae* transmission in Canada. With increasing gonorrhea resistance, emerging preventive interventions, such as doxycycline post-exposure prophylaxis, may need to be considered and universal use of higher doses of ceftriaxone for empiric treatment may become necessary.
